# Supplementary material for: Modeling glioblastoma heterogeneity as a dynamic network of cell states
Source: Mol Syst Biol. 2021 Sep 16;17(9):e10105. doi: 10.15252/msb.202010105 (PMC8444284; doi:10.15252/msb.202010105)
Supplement: Supplementary file 6 — Source Data for Figure 5 [file MSB-17-e10105-s004.zip › Figure5A_sourcedata/GSEA_3017/nosigdb_stateA.GseaPreranked.1622795026485/SEURAT_CELLCYCLE.html]

Details for gene set SEURAT\_CELLCYCLE[GSEA]

|  || Dataset | state53017 |
| Phenotype | NoPhenotypeAvailable |
| Upregulated in class | na\_pos |
| GeneSet | SEURAT\_CELLCYCLE |
| Enrichment Score (ES) | 0.5278051 |
| Normalized Enrichment Score (NES) | 3.2691185 |
| Nominal p-value | 0.0 |
| FDR q-value | 0.0 |
| FWER p-Value | 0.0 |
Table: GSEA Results Summary

  

Fig 1: Enrichment plot: SEURAT\_CELLCYCLE      
 Profile of the Running ES Score & Positions of GeneSet Members on the Rank Ordered List

  

| PROBE | GENE SYMBOL | GENE\_TITLE | RANK IN GENE LIST | RANK METRIC SCORE | RUNNING ES | CORE ENRICHMENT || 1 | CDK1 |  |  | 3 | 1.006 | 0.0442 | Yes |
| 2 | MKI67 |  |  | 4 | 0.926 | 0.0879 | Yes |
| 3 | KIF11 |  |  | 6 | 0.870 | 0.1279 | Yes |
| 4 | UBE2C |  |  | 7 | 0.793 | 0.1652 | Yes |
| 5 | KIF20B |  |  | 10 | 0.749 | 0.1984 | Yes |
| 6 | CKS2 |  |  | 40 | 0.587 | 0.1952 | Yes |
| 7 | HMMR |  |  | 43 | 0.559 | 0.2194 | Yes |
| 8 | HJURP |  |  | 47 | 0.539 | 0.2416 | Yes |
| 9 | CKS1B |  |  | 50 | 0.530 | 0.2645 | Yes |
| 10 | CENPE |  |  | 62 | 0.496 | 0.2762 | Yes |
| 11 | TPX2 |  |  | 66 | 0.488 | 0.2960 | Yes |
| 12 | DLGAP5 |  |  | 76 | 0.475 | 0.3088 | Yes |
| 13 | AURKA |  |  | 78 | 0.474 | 0.3301 | Yes |
| 14 | CDC20 |  |  | 80 | 0.472 | 0.3513 | Yes |
| 15 | TUBB4B |  |  | 93 | 0.462 | 0.3603 | Yes |
| 16 | CENPF |  |  | 94 | 0.461 | 0.3820 | Yes |
| 17 | TOP2A |  |  | 95 | 0.457 | 0.4035 | Yes |
| 18 | AURKB |  |  | 108 | 0.446 | 0.4118 | Yes |
| 19 | CDCA8 |  |  | 115 | 0.437 | 0.4260 | Yes |
| 20 | KIF23 |  |  | 116 | 0.436 | 0.4466 | Yes |
| 21 | HELLS |  |  | 135 | 0.417 | 0.4470 | Yes |
| 22 | GTSE1 |  |  | 146 | 0.411 | 0.4558 | Yes |
| 23 | CCNB2 |  |  | 147 | 0.411 | 0.4752 | Yes |
| 24 | ANLN |  |  | 152 | 0.407 | 0.4901 | Yes |
| 25 | KIF2C |  |  | 165 | 0.397 | 0.4961 | Yes |
| 26 | NDC80 |  |  | 173 | 0.393 | 0.5071 | Yes |
| 27 | BUB1 |  |  | 188 | 0.384 | 0.5103 | Yes |
| 28 | CDCA3 |  |  | 203 | 0.372 | 0.5130 | Yes |
| 29 | SMC4 |  |  | 217 | 0.367 | 0.5164 | Yes |
| 30 | CENPA |  |  | 243 | 0.352 | 0.5064 | Yes |
| 31 | FEN1 |  |  | 259 | 0.343 | 0.5066 | Yes |
| 32 | TTK |  |  | 262 | 0.340 | 0.5205 | Yes |
| 33 | CLSPN |  |  | 274 | 0.336 | 0.5246 | Yes |
| 34 | G2E3 |  |  | 302 | 0.325 | 0.5112 | Yes |
| 35 | ECT2 |  |  | 365 | 0.305 | 0.4595 | Yes |
| 36 | CDCA2 |  |  | 369 | 0.304 | 0.4707 | Yes |
| 37 | CKAP2 |  |  | 379 | 0.301 | 0.4753 | Yes |
| 38 | RRM2 |  |  | 383 | 0.300 | 0.4862 | Yes |
| 39 | CDC6 |  |  | 391 | 0.297 | 0.4927 | Yes |
| 40 | HMGB2 |  |  | 424 | 0.288 | 0.4722 | Yes |
| 41 | NUSAP1 |  |  | 430 | 0.286 | 0.4804 | Yes |
| 42 | NUF2 |  |  | 434 | 0.285 | 0.4907 | Yes |
| 43 | NCAPD2 |  |  | 440 | 0.283 | 0.4987 | Yes |
| 44 | CDC25C |  |  | 443 | 0.282 | 0.5098 | Yes |
| 45 | CDC45 |  |  | 446 | 0.282 | 0.5210 | Yes |
| 46 | ANP32E |  |  | 453 | 0.280 | 0.5278 | Yes |
| 47 | ATAD2 |  |  | 470 | 0.275 | 0.5237 | No |
| 48 | BLM |  |  | 490 | 0.272 | 0.5163 | No |
| 49 | TMPO |  |  | 584 | 0.253 | 0.4292 | No |
Table: GSEA details [plain text format]

  

Fig 2: SEURAT\_CELLCYCLE: Random ES distribution      
 Gene set null distribution of ES for **SEURAT\_CELLCYCLE**
